# Supplementary figures and images for: Mechanics of lung cancer: A finite element model shows strain amplification during early tumorigenesis
Source: PLoS Comput Biol. 2022 Oct 24;18(10):e1010153. doi: 10.1371/journal.pcbi.1010153 (PMC9632844; doi:10.1371/journal.pcbi.1010153)

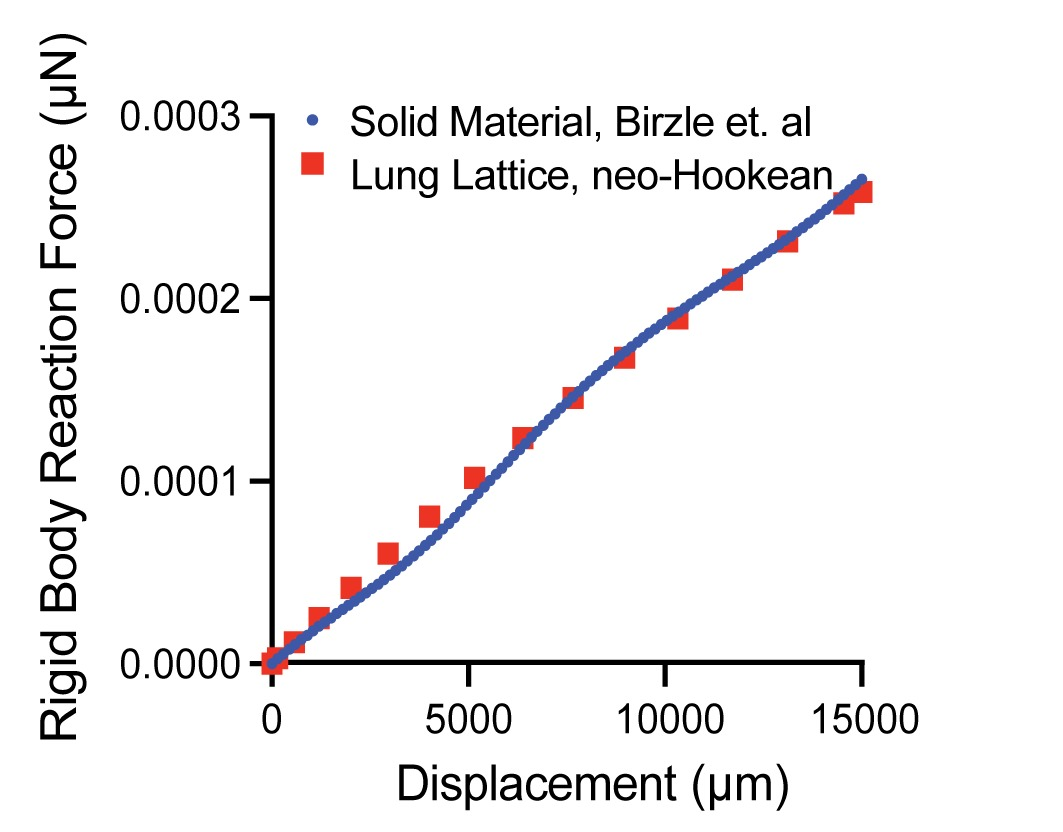

Supplement: S1 Fig — Force-Displacement relationship of the Birzle, et al. constitutive model [43] and the neo-Hookian approximation (Modulus = 35 kPa, Poisson Ratio = 0.25). The assessed range of displacement values reflected physiological strains. Displacement of 15,000 μm = 50% applied stretch. Displacement of 15,000 μm = 50% applied stretch. (TIF) [file pcbi.1010153.s001.tif]

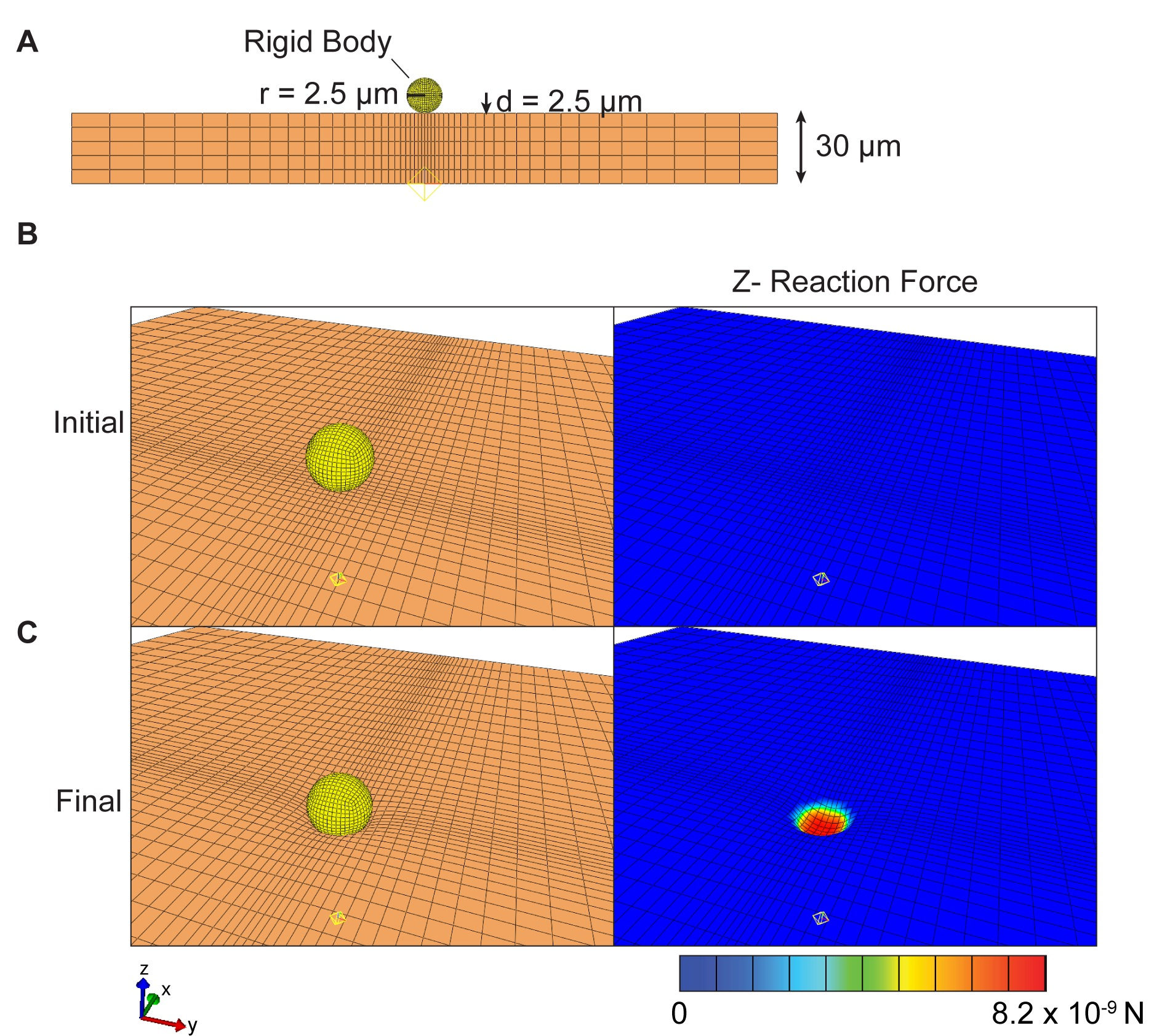

Supplement: S2 Fig — A. Schematic of simulated indentation. A spherical rigid body represents the AFM tip and a solid volume matches the Birzle, et al. constitutive model [43]. The rigid body is displaced vertically at an indentation depth (d) equivalent to the sphere radius (r), 2.5 μm. B. Visualization of the rigid sphere and associated reaction force in the initial position above the material and C. after displacement into the material. (TIF) [file pcbi.1010153.s002.tif]

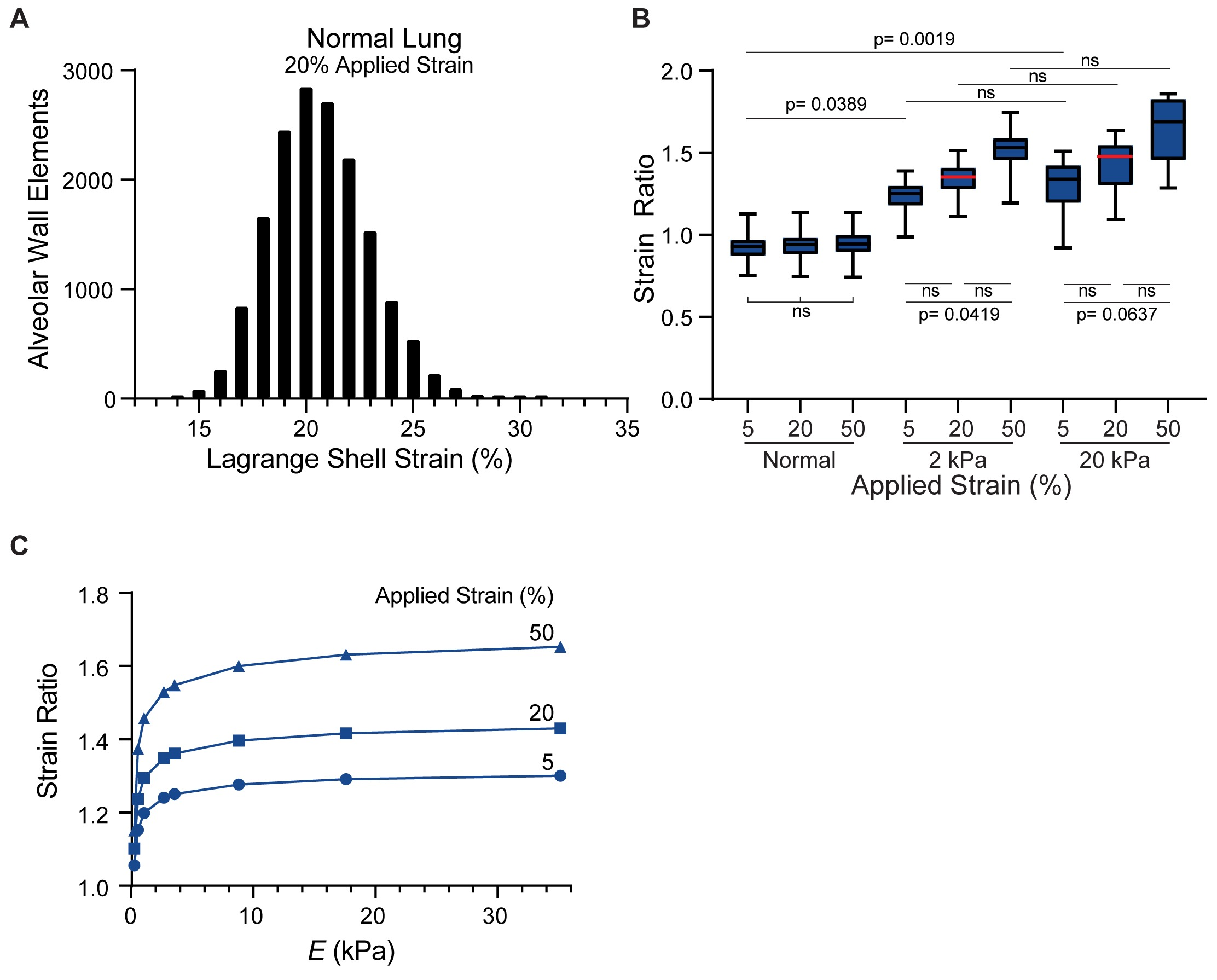

Supplement: S3 Fig — A. Distribution of alveolar wall strain in the normal lung model (no tumor) under 20% applied stretch. The 8 layers of alveoli around the edge of the modeling domain were excluded from the calculations to avoid edge effects. Median Lagrange shell strain is 20.51%, which corresponds to a strain ratio of 0.93. B. Strain ratio in the tumor-adjacent alveolar walls (Fig 3D–3F wall 1). Boxes are 25th percentile to 75th percentile with the median marked by the central line. Red lines show the median Lagrange shell strain for the 2 kPa and 20 kPa tumors at 20% stretch (29.8% and 32.5%), which correspond to strain ratio 1.4 and 1.5. C. Relationship between tumor modulus and strain ratio for applied stretch values of 5, 20, and 50%. The strain ratios for large tumor modulus values converged at 1.7 with 50% stretch, 1.5 with 20% stretch, and 1.3 with 5% stretch. (TIF) [file pcbi.1010153.s003.tif]

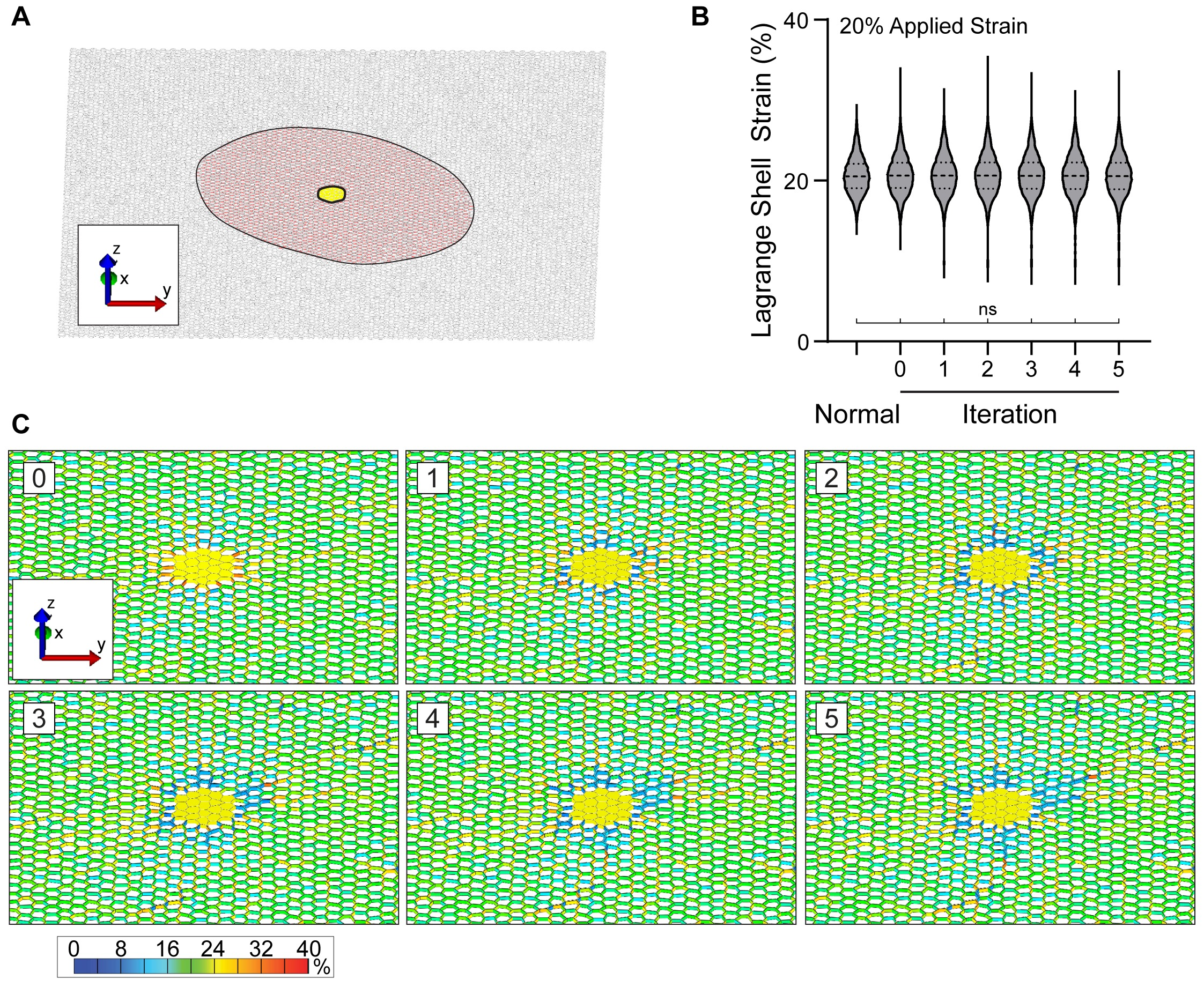

Supplement: S4 Fig — A. Region of interest including 5,432 elements selected for analysis around the tumor includes ~15 layers of alveoli. B. Distribution of Lagrange shell strain in all selected elements in the normal lung and after each simulation iteration of 20% applied stretch and shell-thickening in Fig 6A). C. Colormaps of Lagrange strain in alveolar walls around the tumor during strain-mediated thickening. Lattice is positioned to match the orientation of the tumor in Fig 6A. (TIF) [file pcbi.1010153.s004.tif]
