# Supplementary material for: Mechanics of lung cancer: A finite element model shows strain amplification during early tumorigenesis
Source: PLoS Comput Biol. 2022 Oct 24;18(10):e1010153. doi: 10.1371/journal.pcbi.1010153 (PMC9632844; doi:10.1371/journal.pcbi.1010153)
Supplement: S1 Table — (DOCX) [file pcbi.1010153.s005.docx]

**S1 Table: Mesh Convergence Values**

| **Number of Elements** | **Tumor diameter**  **at 50% stretch** |
| --- | --- |
| 3860 | 67.49 |
| 3860 (2^nd^ Order) | 67.103 |
| 11829 | 69.884 |
| 47231 | 70.289 |
