# Supplementary material for: Mechanics of lung cancer: A finite element model shows strain amplification during early tumorigenesis
Source: PLoS Comput Biol. 2022 Oct 24;18(10):e1010153. doi: 10.1371/journal.pcbi.1010153 (PMC9632844; doi:10.1371/journal.pcbi.1010153)
Supplement: Attachment — Submitted filename: LungMechanicsPLoSCoverLttr-Resubmit2.pdf [file pcbi.1010153.s006.pdf]

HUNTSMAN CANCER INSTITUTE
UNIVERSITY OF UTAH
HEALTH
UNIVERSITY OF UTAH

Oncological Sciences
2000 Circle of Hope, Room 3722
Salt Lake City, UT 84112
801-585-7774

PLOS Computational Biology
ploscompbiol@plos.org
April 27, 2022

Dear Editors,

Enclosed please find our revised manuscript "*Mechanics of lung cancer: A finite element model shows strain amplification during early tumorigenesis*." We are enthused to see that the impact of our study was appreciated.

We thank the reviewers for identifying some areas in which we could improve the communication of our model and findings. We have addressed each point as described below.

Reviewer #1:
1. A brief review of the literature on computational models of lung mechanics and lung tumors should be added to the Introduction. The novel contribution of this work to the field should be better highlighted by clearly differentiating it from previous publications, especially reference [37].
The original reference [37] is now reference [43]. *The following discussion has been added to the introduction:*

"Previous lung models have characterized the material behavior and geometric distortions of the lung during respiration and fibrosis. Early constitutive lung models described the uni-axial stretch of bulk lung tissue [36–38]. Early network models with lattices of spring elements revealed the relationship between lung pressure and volume [39,40]. They showed that alveolar wall stress and stiffness control lung elasticity [39,40]. More recently, a geometric study of lung distortion showed that uni-axial stretch causes the alveolar walls to align parallel to the axis of stretch, rather than elongate [41]. The more physiological elongation-type deformations were captured by new constitutive models that applied bi-axial stretch to decellularized tissue [42] and incorporated pressure-volume changes in bulk lung [43]. While the model by Birzle et. al. described native lung behavior [43], it did not allow for the investigation of heterogeneous alveoli deformation."

2. Why is FEBio software chosen for this study? Please provide the most relevant feature(s) of this software that contributed in deciding to use it here.

FEBio was chosen for its utility in solving non-linear large deformation problems and ease of assigning material properties to individual parts of a complex geometry. Since FEBio is open source and one of the corresponding authors leads the software project, we were able to easily extend the software to implement the Birzle constitutive model. Use of FEBio and FEBio Studio also makes it straightforward for us to share the models used in this research after publication via the model repository that is integrated with FEBio Studio. A statement to this effect has been added to the Methods - Description of the finite element models of lung tissue.

3. In the subsection titled 'Description of the finite element models of lung tissue', the following statement is made: 'To account for variability in lung architecture, a randomization algorithm was applied to the initial geometry, followed by simulated annealing through energy minimization [38]'. How does this work exactly? A very brief description of this process (either in the text or as a footnote) would help.
The following description has been added to the Methods subsection:

"Briefly, the nodes of the hexagonal units were subjected to a random displacement (alpha = 0.1) followed by rotation of the resulting hexagons (beta = 4.21) such that the resulting aspect ratio was ~0.2. MATLAB software for the randomization is shared via GitHub at https://github.com/MendozaLabHCI/TumorLungMechanics_2022."

4. From the text, it is not clear which of the two strain energies (1) or (3) are used to model the healthy lung tissue and the tumor. Is the so-called Birzle hyperelastic constitutive model (1) used for the lung tissue without tumor and for the lung tissue surrounding the tumor, while the compressible neo-Hookean hyperelastic constitutive model (3) is used only for the tumor? Or model (3) is used to model the mechanical behavior of both lung tissue and tumor in the case that the tumor is present? Please clarify and make necessary edits to the test accordingly.
We appreciate the reviewer bringing to our attention that this was not clear. We used the Birzle hyperelastic constitutive model (1), which describes the bulk lung behavior, to define the alveolar wall behavior. We used a compressible neo-Hookean strain energy to describe the alveolar walls in the normal lung and the tumor. To get from the bulk lung behavior (1) to the behavior of alveolar walls (3) we used parameter optimization as described in the methods section "Parameter optimization based on bulk lung constitutive model."

We modified the Methods subsection to clarify our approach:

"To ensure that the constitutive model and associated material coefficients that we used for the alveolar walls provided an accurate representation of lung tissue material behavior, we implemented a hyperelastic constitutive model in FEBio that was developed previously by Birzle et al. to describe the continuum level behavior of bulk lung tissue [37]. To determine the material behavior of the alveolar walls in our lattice geometry, we fit the material coefficients of a neo-Hookean hyperelastic constitutive model to the behavior predicted by the Birzle bulk lung constitutive model.

5. What boundary conditions were used on the lung tissue without a tumor? What extra

boundary conditions were imposed in the case of the lung tissue with a tumor at the interface between the tumor and the surrounding tissue?

We have clarified this in Methods subsection - Description of the finite element models of lung tissue:

“Stretch was applied on the 4 boundaries in x and y to obtain the prescribed elongation. Plane strain boundary conditions were assigned to both the non-tumor and tumor-containing models. Thus, equibiaxial elongation was applied along the x and y directions while their geometry was constrained in the z-direction.”

6. *The statistics was calculated with the Andersen-Darling, nonparametric Kruskal-Wallis, and post-hoc Dunn’s multiple comparisons tests. What are the most important features of these tests that contributing in deciding to use them here? (This could be a footnote.)*

We have updated the Methods subsection- Statistics to read:

“Not all data sets were normally distributed, so non-parametric tests were employed. For comparison of mean alveolar wall widths, significance between the two groups was tested using the Mann-Whitney test. For comparison of mean strain ratios, significance was tested with the Kruskal-Wallis test, which allows comparisons of three or more means. To determine which means were different from each other, a post-hoc Dunn’s multiple comparisons test was used..”

7. How do Figs. 6 D-E compare to Fig. 6 A? Highlighting the tracks mentioned in the caption of Fig.6 on the images in Fig. 6 D-E with a marker (instead of just showing stars and arrows) might help the comparison with the finite element results shown in Fig. 6 A.

In order to make the tracks easier to evaluate, in Figure 6 D, a replicate image annotated with a red line overtop of the matrix tracks has been included. The legend has been modified accordingly.

8. Are there any special characteristics that a cancerous lung tumor has that makes this study work only for these tumors and not for benign ones? For instance, how do the cells geometry look in a cancerous lung tumor and its surrounding tissue? Are there any differences seen in the cells geometries in a healthy lung tissue versus a lung tissue with a cancer tumor present? How do these shapes compare to those for a benign tumor? Could this be expected to happen in the case of a benign tumor? Given the diffusive character of cancer cells that makes them so invasive, could a connection between the tracks shown in Fig. 6A and the possible diffusion paths taken by the cancer cells be established? Lastly, could it be possible that at a certain strain threshold, a certain mechanotransduction process be activated that will transform a benign tumor into a cancer one? Some of these questions go beyond the work presented here so they do not need to be addressed.

We are enthused to see that the reviewer is interested in the significance of our model. The special characteristic of cancerous lung tumor that is included in the current model is tumor stiffening. The model shows that stiffening exacerbates the strain amplification. The inclusion of other characteristics and testing of mechanotransduction in the transformation processes are the focus of our future work.

We have addressed the questions about benign tumors by including the following statements in the discussion:

"We expect that some strain amplification would also occur with benign lung tumors, although to a lesser extent since benign tumors exhibit abnormal growth and thickening but retain an alveolar structure… The *in vivo* consequence of strain to the disease progression of benign and cancerous lesions remains to be determined."

Our original discussion addressed the connection about tracks and invasion, "Tracks of aligned ECM, including those composed of collagen, fibronectin, and Tenascin-C are a signature of invasive cancer and can serve as a conduit for tumor cell dissemination [49,51,53,64–66]. Tumor cells preferentially invade along aligned fibers and we observed tumor cells populated along Tenascin-C tracks in early human LUAD. Together, these results suggest that strain amplification in early lung cancer may initiate Tenascin-C-containing matrix deposition, which creates tracks of matrix that promote tumor cell invasion."

Elucidating how these paths enable tumor cell invasion in lung cancer requires live-cell imaging and is out of the scope of this work.

Reviewer #2:

1. - how do they implement the stretching of the tissue, i.e. did all boundaries move or just top/bottom?

This has been clarified in the methods with the addition the following statement:

"Stretch was applied on the 4 boundaries in x and y to obtain the prescribed elongation."

2.- would the choice of geometry affect the results if a radial geometry was used instead of a cartesian setup?

No, we do not expect that there would be a difference if a radial geometry was used instead of the cartesian coordinate system. In both coordinate systems we would expect the nodes in the center of our tissue to move equivalently under deformation. We chose the cartesian coordinate system because it is more straightforward to compute the strain along alveolar walls across the whole tissue.

3. - does the tissue experience any shear around the tumor?

Yes, the alveolar walls around the tumor experience some shear strain. The text in the methods has been modified to clarify this and now includes the following statement:

"The applied strain induced shear strain and first principal strain. We focused on the local first principal strain to understand how the alveolar walls stretch in the absence and presence of a tumor."

4.- is lung tissue generally isotropic or anisotropic? Are there implications of this assumption on their results?

Lung parenchyma is generally considered to be isotropic with no preferred fiber direction at the scale of the bulk lung tissue as well as at the scale of the alveolar wall. We addressed this by adding the following statement to the discussion:

“Lung parenchyma is homogenous and isotropic [75], therefore the strain energy function derived from bulk material characterization is applicable to our lung lattice model without consideration of tissue orientation.”

5. - what are the limitations of the 2D model compared to the 3D lung?
We have addressed this limitation by adding the following statement to the Discussion:

“A limitation of the 2.5D model, compared to the 3D lung, is that the junctions between the alveoli and the top and bottom surface of the alveolar sheet are modeled as uniformly perpendicular to the plane of the sheet. This restricts the angle of strain with respect to the tumor, and may predict more uniform tracks than might be observed with a randomized 3-dimensional geometry.”

6. - should equation 5 be solved for E if that is the formula used to compute E from F?
Equation 5 (the Hertz model) has been rearranged and now reads: $E = \frac{3\,F\,(1-\nu^2)}{4(\sqrt{Rd^3})}$

Data availability:

The MatLab code used to construct the lung lattice geometry has been uploaded to a publically available repository: (https://github.com/MendozaLabHCI/TumorLungMechanics_2022). FEBio is open-source and freely available at https://febio.org/. The bulk lung constitutive model from Birzle et. al. is available as a material in FEBioStudio.

We are very grateful for the suggestions and opportunity to improve our manuscript. We look forward to your positive response and our work’s dissemination within the lung mechanics and cancer biology communities!

Yours Sincerely,

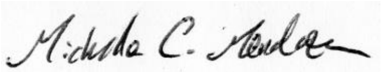

Michelle C. Mendoza, Ph.D.
Assistant Professor in Oncological Sciences
Huntsman Cancer Institute, University of Utah
